# RecMem: Time Aware Recommender Systems Based on Memetic Evolutionary Clustering Algorithm

**DOI:** 10.1155/2022/8714870

**Published:** 2022-04-07

**Authors:** Raheleh Ghouchan Nezhad Noor Nia, Mehrdad Jalali

**Affiliations:** ^1^Department of Computer Engineering, Mashhad Branch, Islamic Azad University, Mashhad, Iran; ^2^Institute of Functional Interfaces (IFG), Karlsruhe Institute of Technology (KIT), Hermann-von Helmholtz-Platz 1, Eggenstein-Leopoldshafen 76344, Germany

## Abstract

Nowadays, the recommendation is an important task in the decision-making process about the selection of items especially when item space is large, diverse, and constantly updating. As a challenge in the recent systems, the preference and interest of users change over time, and existing recommender systems do not evolve optimal clustering with sufficient accuracy over time. Moreover, the behavior history of the users is determined by their neighbours. The purpose of the time parameter for this system is to extend the time-based priority. This paper has been carried out a time-aware recommender systems based on memetic evolutionary clustering algorithm called RecMem for recommendations. In this system, clusters that evolve over time using the memetic evolutionary algorithm and extract the best clusters at every timestamp, and improve the memetic algorithm using the chaos criterion. The system provides appropriate suggestions to the user based on optimum clustering. The system uses optimal evolutionary clustering using item attributes for the cold-start item problem and demographic information for the cold start user problem. The results show that the proposed method has an accuracy of approximately 0.95, which is more effective than existing systems.

## 1. Introduction

While the Internet is useful, it causes problems to users. There is a huge amount of information that makes it difficult for users to find relevant, appropriate and precious knowledge. In general, the recommender systems are divided into two categories: collaborative filtering (CF) and content filtering. Content filtering compares the similarity between a suggested item and an item that the target user likes or dislikes. The comparison is based on the features of the item and its rates. CF makes it possible to find users with similar interests to the target user. The CF is based on previously assigned ratings for a particular item.

The preference of users who work with the system changes over time, and an item that was interest to the user over a period of time may no longer be interest in it. The system should consciously perform the evolutionary clustering over time so that it can apply changes of style. Existing systems do not use the memetic algorithm to extract the optimal clustering, nor do they address cold start problems using this algorithm [1]. A cold start problem is an issue that has just logged in and has no history of behavior in the system or item rates [1].

If the user becomes interested in another item gradually by clustering at the present moment regardless of the time dimension; the system cannot affect these changes in clustering; therefore, it makes irrelevant suggestions to the user [2]. The most important challenge is to improve system accuracy by changing user requirements and content ratings. Traditional recommendation systems cannot make changes according to user preferences. Many problems are solved by considering the time dimension in the recommender systems. In [3], a new algorithm based on the Memetic and Metaheuristic algorithms is proposed, which is enhanced by the simulated annealing algorithm but does not take into account the time parameter.

Furthermore, [4] demonstrated how changes over time affect the accuracy of the system. Because time is not yet a challenge in evaluating and analyzing CF, few people relate the impact of the time factor to the recommendation process, but evolutionary algorithms with the application of time dimension provided good development in the recommendation process. The concept of evolutionary clustering is presented in [2]. Clustering can be very effective in identifying user similarity and optimal clusters improve the accuracy of suggestions. The evolutionary clustering approach can be used in recommender systems to combine the idea of changing user interests the passing of time and system content. This paper presents a recommender algorithm based on memetic evolutionary clustering that can improve accuracy and deal with the cold start problem. Moreover, the time dimension can also increase the scalability of the system. The CF has been used in numerous recommender systems, leading to information filtering and accurate information selection in the database. Collaboration is defined as information prepared by all system's users [5]. In general, the scalable algorithm is applied in CF recommender systems to integrate new data over time and update the current state of the user profile. This study demonstrates the effectiveness of the evolutionary memetic clustering algorithm, which can take into account different conditions with pass time and apply the necessary changes. The chaotic criterion is used to enhance the performance of the memetic algorithm in order to optimize clusters more accurately. The evolution of clusters looks at user preferences at various times. This method uses a score called a variance score to compute the difference between user scores and, ultimately, to contemplate changing the evolutionary process at any time. The proposed approach is a recommendation system that groups users at different times and applies their interest variations. Clustering at each timestep is evaluated by two parameters of clustering quality and their differences with previous clusters. At each timestep, the quality of the clustering should be high and the difference with the previous clustering should be low.

The rest of the paper is as follows: in Section 2, the literature is reviewed.  Section 3 is discussed the proposed method.  Section 4 is evaluated the results. Discussion and conclusion are provided in Section 5. Future work will be discussed at Section 6.

## 2. Literature Review

The temporal rule mining model developed to extract important sequential patterns containing not only the recommended items but also the time gaps among them [6]. Sequential patterns are effectively extracted by an evolutionary-based method called Genetic Network Programming (GNP) [7]. The if–then connection in time-related association rules has similar features to the directed connection structure in the ant colony optimization (ACO) method which is proposed by Merkle [8]. High-order star structured coclustering techniques have been used by some authors previous work [9, 10] to solve the problem of prefiltering heterogeneous data [11]. In [12, 13] is proposed a method to combines the low-level and high-level features of items which the possible past behavior of users and general behavior of all users in the community is provided useful recommendations during the browsing multimedia collections [11].

Hartigan and Wong combined K-means clustering [14] and a variant of Particle Swarm Optimization [15] to divide users into the optimal clusters. It is included predicting the missing ratings of web services that have not been recalled by similar users, i.e., consumers activate in the same fields as the current user [16]. To predict the missing ratings, they use the Slope One method [17] based on time factor [16]. A new genetic algorithm encoding is proposed as an alternative of k-means clustering [18]. The introductory problem in classic k-means is targeted by proposing a formula of the problem to reduce the impact of the search space complexity as well as to improve clustering quality [18]. The bio-based clustering is presented through the integration of swarm intelligence and fuzzy clustering models for user-based CF [18]. The CF algorithm faces various complications in the rating prediction process due to data deficiencies or lack of ratings. Cosine similarity performance is reduced by identifying similar locations and users due to the lack of data problem [19]. Bio-Inspired Clustering Ensemble (BICE) based on Collaborative Recommender System (CFRS) performs neighbor search according to the active target user to fit the user in the cluster truely [19, 20]. In [20], they apply knowledge-based evolutionary optimization algorithms called Cultural Algorithms (CA) to assess similarity between users. To deal with the sparsity of data, they combine CF with a trust network between users [20]. The trust network is then clustered using Singular Value Decomposition (SVD) which helps to discover the highest level of neighbor's trust [21]. They chose Singular Value Decomposition (SVD) to cluster trusted communities into the different groups [21]. In [22], they propose a deeper graph neural network that can predict links on a bipartite user-item graph using information propagation. It is solved the input of variable size for each node in a bipartite graph [22]. In [23], they showed that the mathematical expression is determined as follows: (1) the conversion of some visual and qualitative conditions, which should be satisfied by similarity criteria to the corresponding mathematical equations: integral equation, linear system of differential and nonlinear equations, and (2) Solve equations to achieve similarity core performance measurement. The social recommender system is proposed based on reliable implicit relationships [24].

The Dempster-Shafer theory is used as a powerful mathematical tool to calculate the implicit relationships [24]. Criteria for assessing the reliability of predictions were introduced, while unreliable predictions were recalculated using the neighborhood improvement mechanism [24]. The mechanism uses inter-user confidence coefficient to identify ineffective users in the target user's neighborhood and recalculates new reliable ratings [24]. The social regularization combination approach that incorporates social network information to benefit recommender systems with the trust information between users [25]. Trust and rating records are used to predict the missing values in the user-item matrix, using an algorithm to best select the recommended trust path to identify several recommended trust paths and determines a universal path for generating various final recommendations and it can deal with cold start problem [25]. In each user group, user correlation is applied to choose the nearest neighbors to predict ratings [26]. The Collaborative Filtering Recommendation Algorithm Based on User Correlation and Evolutionary Clustering (denoted as UCEC&CF) algorithm is proposed [26]. The hierarchical hidden Markov model uses to identify changes in user's preferences over time by modeling the latent context of users [27]. Using the user-selected items, the user considers as a hidden Markov process and the user's current context as a hidden variable [27]. The latent contexts are automatically learned for each user utilizing hidden Markov model on the data collected from the user's feedback sequences [27]. This classification is carried out in order to reduce the overall operating cost and to satisfy the minimum up/down constraints easily [28]. One method is used the concept of clustering algorithm as a hierarchical clustering based on the hybrid technique of genetic algorithm and simulated annealing in order to perform the unit commitment technique [28]. Four techniques formed through subgroups of correlated items based on a set of similar users to be predicted only for related items [29]. Because users in each subgroup have similar preferences in the item subgroup, they explore different ways to select user-item subgroups with high correlation to predict user ratings for the unseen items [29]. The deep learning hybrid recommender system eliminates gaps in CF systems and achieve the most advanced and accurate predictions using deep learning [30]. The solution is benchmarked against existing methods on both predictive accuracy and running time [30]. Uses these interpretations to learn nonlinear latent factors for users and items, and combines deep learning features with existing information about users and items to create a hybrid system based on learning rates and weighting [30]. An integrated recommender approach based on network science tools has solved the problem of data sparsity [31]. The link prediction approach is used to extract hidden structures among users, and diffusion of information is applied to enhance the rating matrix [31]. The sparsity problem is solved through a more efficient way, and the hybrid method can also be used with well-known algorithms [31]. The aim is to enhance the similarity computation among users by applying the meta-paths, also, a link prediction method is applied to discover the hidden connections among users to relieve the sparsity problem [31]. The diffusion rules are applied to predict the hidden ratings with the propagation among similar users in the built-up network [31]. A method using network prediction tools such as link prediction and propagation rules to solve the sparsity problem of user-item matrix [31]. The architecture allows the recommender system to utilizes rich data collected about the user to produce more accurate recommendations, while allowing its users to manage and control their data [32]. From the user's point of view, sending data to the server and the user's inability to track and control the data leads to repetition of the process with multiple servers and this causes the data to be stored and accessed in different servers without the user's notice [32]. An attention-based context-aware sequential recommender model is proposed using Gated Recurrent Unit (GRU) as ACA-GRU in [33]. The impact of information content on recommendations has been examined and classified into four categories, which include input content, content correlation, the concept of stable interest, and transition content [33]. By redefining the updating gate of the GRU unit, the sequential state transition of the RNN determined by these concepts has been calculated to model the dynamics of user interest and the context correlation mechanism, which is able to distinguish the importance of each item in the rating sequence [33]. Microblogging systems need to predict and understand user interests to provide more products, advertising, and personalized services. However, identifying user's interest and preparing personal suggestions to the user is still a major challenge [34–36]. Microblog contents has general feedback on personalization. Distributing content on the microblog creates problems for suggestions [36, 37]. The tags that users tag for content use user-tag matrix to solve the distribution problem, while user-user matrix may precisely cluster users and make appropriate recommendations [36].

The user performance model requires feedback from other users or current user to give appropriate recommendations to the user and no longer pay attention to the ratings they give over time [38]. These scores change over time and do not require system learning, but rather puts them in new clusters with similar users. It has used evolutionary algorithms to improve the recommender technique, which does not improve clustering [38]. Users are clustered using the memetic algorithm without the chaos criterion. And it does not use demographic information to deal with cold start problem, but uses the text of user's tags. [39]. The Group lens and Ringo systems were the first systems to use the collaborative filtering algorithm. Group lens recommends Usenet papers based on user ratings and user-to-user relation as a measure of similarity. The system was a paper recommender for e-communities, especially UseNet news, and presented two free services for the UseNet environment: the first service provided personal predictions in which the user rated the paper, comments are compared with other people in the group. The system will check the user's performance by their rating [40].

The recommender systems are designed to predict the interests of users. The user profile is the core of these systems. How profiles are created, system default profiles, profile updates, and the source of the updates used in the suggestions are important factors in the performance of the recommender system [5]. Due to the variety of algorithms used in these systems, different categories are proposed in this area. Most recommender systems use collaborative systems to find similar users and the target user's purchase records. A list of recommendations is then prepared by gathering information about the user's favorite items and deleting items that have not been previously purchased by the user. This group of systems which are among the most widely used systems to generate user recommendation [5]. The main mechanism of the collaborative filtering algorithm is that by using the user similarity criteria, the preferences of large groups of users are recorded. Users who have the same priorities with the current user are selected as their neighbors. Then the average priorities are calculated and the final priority function tries to recommend an item that the user has not rated [5]. Content filtering Systems are rooted in the data retrieval. The content-based approach deals with the subject of recommendation to find similar items. Based on purchased and rated items, the algorithm tried to find suggestions with similar keywords and content. Systems implement on a content-based algorithm, given information about users' interests after a matching action, they recommend items with similar content to users' interests [41]. Knowledge-based systems offered based on their perceptions of user needs and item features. In other words, in this type of recommender systems, the raw materials used to generate a list of recommendations are system knowledge about the user and the item [41]. Each customer may purchase a specific item every few years, so the recommender system cannot create more user details, including quality and technical features. As a source of additional knowledge, the system needs to extract additional knowledge to generate recommendations. The knowledge-based recommender system often uses knowledge and reasoning to make suggestions. Knowledge-based recommender systems include constraint-based and position-based [42]. Hybrid recommender systems used combination methods to achieve better comprehensive suggestions. Hybrid methods increase system performance and reduce system disadvantages. Often collaborative filtering approach is one of the hybrid methods. When users have fewer common items, the hybrid method performs better than basic methods [42].

Time-aware recommender system provides a way to integrate social and temporal information into a user-base recommender system [43]. One of the similarity criteria is the simultaneous use of the system by users, i.e., users who visit the system within a week, are identified as similar users. It ignores user rating and creates a model based on conceptual information and user behavior time. [43]. A time-based approach has been proposed that is based on user feedback data and has been collected over time. It is also assumed that users' shopping interests are time-sensitive and that the recommender system is more likely to influence new data from user feedback [44]. Items that have recently been added to the system and displayed for sale to the users can cause the user to be curiosity and have more weight so that user does not use the features of the item and builds the model based on user feedback. However, it does not take advantage of user ratings and evolutionary clustering over time [44]. Research has shown that the extraction of temporal concepts is an effective way to enhance the performance of recommender systems. In [45], it focuses on collaborative filtering techniques and the advantages of using conceptual information relevant to each user, such as temporal and common concepts that are interest of most people. The time-aware systems are considered as a subset of concept-based systems. In general, time aware recommender algorithms are divided into two categories: time aware heuristic and time aware model-based methods [45]. In time-aware heuristic methods, items are suggested to users that have similar rates to the target item. This method has already been introduced as the k-method of the nearest neighbor based on the item. In the time-aware based heuristic method, parameters and system data are dynamically updated as data properties change over time [45]. The same classification in [45] applies to time-aware modeling methods. In all classifications of time-aware methods, user rating data model are constructed and considered dynamically in the system, although the time parameter is continuously categorized in a continuous timesteps and time-lapse intervals are also assumed to be dynamic, as the system uses data updates and does not apply evolutionary algorithms to clustering data over time [45]. Collaborative filtering algorithm can help you to predict the user's interests by collecting and analyzing more information about user behaviors, activities and access to user friends. The computer and the web platform allow you to communicate with users' out of your personal relations and use millions of people's opinions rather than making decisions based on the opinions of ten or a hundred friends and relatives. The speed of computers allows us to process these comments in real time and to know what people thinking like us about a particular item that we do not know about it [46].

Collaborative filters are divided into three general categories, memory-based methods that calculate similarities between users and sources using statistical techniques [16]. Model-based methods that use probabilistic models and machine learning and data mining algorithms to examine user history as instructional data and use actual data to suggest to the user. The advantage of the method is that it manages sparse data better than memory-based methods and is scalable in large data, as well as improving its performance in recommendation algorithms. Hybrid methods consisting of model-based and memory-based methods are used to overcome the disadvantages of the method and improve the suggested results [47]. A new algorithm based on Memetic algorithm and metaheuristic methods has been introduces, although it does not consider the time parameter but the simulated annealing algorithm improves performance [3, 48]. While in the proposed method in this paper uses the Memetic algorithm and the time parameter for cluster evolution. The hybrid memetic algorithm, by changing the input space to higher dimensional feature space, can reduce the computation volume with a constant time parameter and cannot improve the clustering over time [3, 48].

As mentioned, in existing recommender systems, a model is developed based on user behaviors, which is not time sensitive [1, 43, 44]. By weighting new item, it makes users curious to visit it which conventional recommender systems do not use evolutionary clustering to evolve clusters over time based on user interest. Since user interest is not constant and time dependent, the importance of evolution increases the efficiency of recommender systems. Also, using demographic information and item attributes for the cold start problem can produce more precise results, because by weighting the new item, if the user visits and dislike it, it would be suggested the inappropriate item to other users [1, 43, 44]. Performs time aware evolutionary clustering using deep tensor learning and considers the necessary changes required at each clustering timestep. The Tensor latent block model used the three-dimensional matrixes that process different relationships between the entities and do not consider the cold start problem [49]. Uses a variety of analytical patterns for recommender systems to provide rule-based recommendations and cannot evolve clustering over time to discern user interests [50]. Feng et al. in [51] proposed an algorithm to extract association rules which is presented the items relation. They did not consider any time-aware clustering for recommender system to apply the users interest variations [51].

Lonjarret et al. in [51] proposed a model which uses the history behavior of user to prepare recommendations. They do not investigate on evolutionary algorithms to evolve the groups of users and items. Panagiotakis et al. in [52] presented a method to improve the performance of recommendation. They do not use evolutionary algorithms to evolve user interest through the time. They do not deal with the cold start problem in user or item problem. Ghazanfar and Prügel-Bennett proposed a hybrid technique to solve the gray-users problem, which improves system efficiency by separating them from other users [54]. They used offline clustering to detect gray-users and did not investigate time-aware clustering to detect changes in users' interests over time [54].

## 3. Proposed Method

The developed Memetic algorithms are evolutionary algorithms that improve their fitness by adopting local search processes (such as hill climbing) on agents. Although the memetic algorithm gives good results, the memetic algorithm approach depends on the details of the problem. In this paper we proposed a method called RecMem. The main structure of the algorithm consists of four principal methods: the initial method for generating the set of initial solutions; Reproduction method in order to generate a set of children from the main criteria (parents); Integration method to create the next generation population (this population is composed the best solutions of parents and children); And finally, the local optimization method to improve current agents.

**Figure 1:**
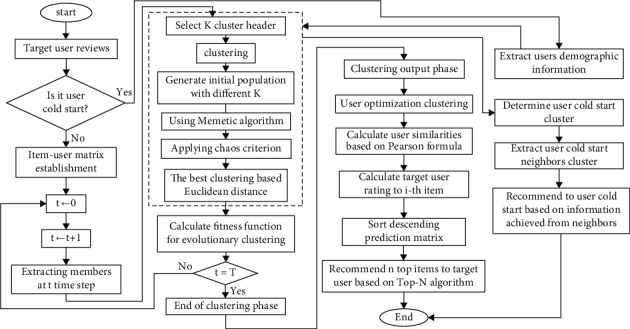
Proposed method chart with cold start user phase.

As shown in Figure 1, after the target user starts, it is checked whether the cold start is or not. If the user is not cold start, the user-item matrix is established at *t* = 0 moment and increase a timestep (*t* = *t* + 1). In a *t* timestep, it extracts members and implements the Memetic algorithm phase. In this phase, it selects the cluster heads to clustering. With different *K*, it creates the initial population and uses the memetic algorithm. The genetic algorithm (GA) is implemented to find the best clustering, and since it cannot find the global optimization, the simulated annealing (SA) algorithm puts on to find the optimal clusters by limiting the search space and extracting the best clustering. It then uses the Euclidean distance to detect the best clustering. The fitness function calculates for all timesteps of evolutionary clustering. By creating the user-item matrix, the time dimension is considered and at each stage of the time it makes the matrix to achieve the optimal clustering and can also make similarities and predictions by using the algorithm and extracting the optimal clustering. It obtains the output of the clustering phase and achieves the similarity of users based on Pearson's similarity. It then predicts the user's rating for the item. Then it arranges the prediction matrix in descending order and offers the top item to the user based on the Top-N algorithm.

The conventional clustering algorithms are not considered in order to group the data according to their temporal dimension. The performance of conventional clustering algorithms got worse across the cluster evolution based on time [1]. The general purpose of any clustering algorithm is to find the clusters that have the best quality which is defined by the fitness function. As a result, finding data clusters with the highest value of fitness function is an optimization problem. Conventional clustering algorithms fail to optimize global fitness function.

**Figure 2:**
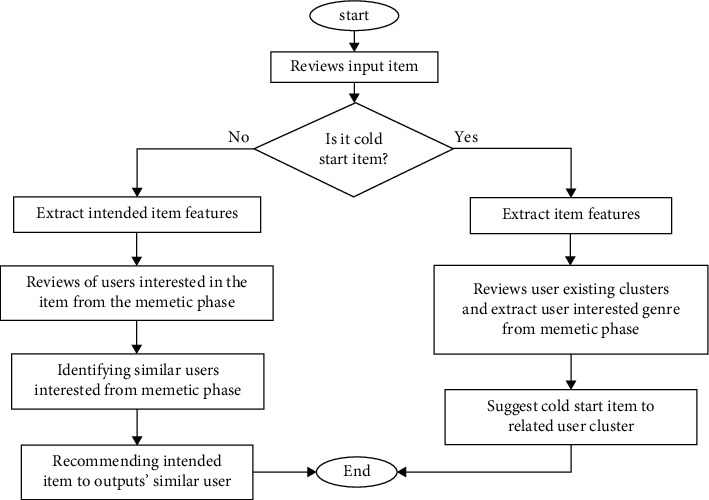
Cold start item phase chart.

The cost function is proposed to optimize the fitness function, which creates accurate clustering for evolutionary algorithms. It firstly checks the user, if the user is not a cold start, the user-item matrix is formed at *t* = 0 and one is added to the time parameter (*t* = *t* + 1). At the timestep *t*, members are extracted and the phase of the memetic algorithm is executed. Selects headers for clusters and is grouped based on them. The initial population is formed with different *K* and finds the best clustering using Euclidean distance. It calculates the fitness function for all timesteps for evolutionary clustering. Once the output of the clustering phase is obtained, the similarity of users is calculated based on Pearson's formula. It is predicted the target user rating to the item. The prediction matrix sorted in descending order. The *N* top items are offered the user based on the Top-N algorithm. Similar users extracted the target user from the memetic algorithm step for clustering items and suggested the target item to similar users. In order to deal with the cold start problem in this paper, user's demographic information is used to determine the cold start user cluster and is recommended to the cold start user based on neighboring information. In Figure 2, it is showed how to cluster a cold start item to offer the cold start item to the users of the relevant cluster. However, if the item entered into the system is cold then item attributes are extracted for cold start item clustering and cold start item is recommended to the relevant users based on existing user clusters and their favorites.

Data stream clustering focuses on optimizing temporal and spatial constraints, while evolutionary clustering depends on time consistency. Therefore, evolutionary clustering is an important field of research that can improve quality in the real world. The evolutionary clustering concepts is focused on the quality of clusters. In memory-based recommender systems, users can usually rate their resources, and recommenders can suggest items based on the most recent items that they have previously rated to improve the quality of the recommendations.

### 3.1. Problem Formulation

Data mining is a combination of different learning algorithms. Clustering algorithms are one of the categories that are mainly used in unsupervised learning. On the other hand, a method called collaborative filtering is used in which suggestions are made by finding similar users and thus predicting new user priorities based on the similarity. Clustering algorithms are an effective application of the CF to find similar for prediction. Also, by changing user needs over time and dealing with the cold start item, a new mechanism is needed to offer users the proper recommendation. To implement such a recommender system, a clustering framework named RecMem is proposed. The evolutionary clustering, presented in [55], did not use the time dimension; in this study, clusters categorize the users by time interval. At each timestep, a new cluster is generated by optimizing two new parameters, called Snapshot Quality (SQ) and History Cost (HC). The SQ depended on the quality of the cluster and its accuracy. The HC indicated how different the new cluster is from the previous one. In this context, the focus is on cluster transfers, which over time are provided by increasing the SQ and decreasing the HC. The sum of the quality of this sequence is shown by

(1) $$\begin{array}{c} \mathrm{fitness}\;\mathrm{function}=\sum_{t=1}^{T}SQ\left(C_{t}M_{t}\right)-\sum_{t=2}^{T}HC\left(C_{t-1},C_{t}\right), \end{array}$$

where *SQ*(*C*_*t*_*M*_*t*_) is the snapshot quality of the *C*_*t*_ cluster at timestep *t* with *m* input and *HC*(*C*_*t*−1_, *C*_*t*_) is the history cost of the *C*_*t*_ cluster at timestep *t* − 1.

The *U* = {1,2,3,…, *n*} is a set of objects that need to be clustered. At each *t* time interval, which is 1 ≤ *t* ≤ *T*, a new dataset is entered. It is assumed that data could be represented by a matrix *M*_*t*_ with size *n∗m*, which is expressed the dependency between each pair of data targets. It is defined evolutionary clusters as a set of items in a *t* time interval. Suppose, *T* = {1,2,3,…, t} is a finite set of time intervals and *I*={*i*_1_, *i*_2_,…, *i*_*tm*_} is a set of *m* items and *I*={*u*_1_, *u*_2_,…, *u*_*tn*_} is set of *n* new users, at different time intervals. So, assume *C*_1_ is the cluster created at time interval *T*_1_; when the new item *i*_*t*_ is added at time interval *t*, or an old item priority level and rating are also different; here the RecMem function produces the new clustering *C*_*t*_, which has optimal clustering quality. A group of clusters are known as *C*_*t*_, where *C*_*t*_={*C*_*t*1_, *C*_*t*2_, *C*_*t*3_,…, *C*_*tk*_}, and *K* is the number of items in the cluster. *C*_*t*_ is the group of items given at each interval and the segmentation of the items must be such that, in each cluster, the highest similarity and the least difference. This goal can be achieved by clustering algorithm and defining the cost function, which refers to the cluster quality. In proposed method, *M*_1_,…, *M*_*t*_ are the input matrices at each time interval *t*, and *C*_1_,…, *C*_*t*_ are the clusters generated at time *t*, which are created based on new matrices and new histories. More various functions have been suggested by different researchers to determine the cost function. To the best of our knowledge, the most helpful of the proposed method is the formation of a cost function, which optimizes two incompatible parameters, which are particularly useful for detecting user interest variations over time. The cost function is a combination of snapshot cost and the history cost, used in [3], which cost function can display history cost and snapshot quality.

The function optimized two competitive goals of SQ and HC. The SQ shows how well the data clusters display over time at timestep *t*. This criterion is defined by using a scale called variance, that minimized the difference between items in each cluster and maximized their similarity. Implementation of variance has been done in [55–58]; in [2], also effective timestep quality has been presented. The variance difference of rates is between item grouping in a particular cluster at a specific timestep. The higher variance, described the low SQ. A time interval *t* has been chosen, which expressed the maximum contribution to this problem. The formulas (2) and (3) are used to calculate these two [1]:

(2) $$\begin{array}{c} SQ\left(C_{t}M_{t}\right)=\sum_{t=1}^{T}\left(1-VScore\left(M_{t},t\right)\right), \end{array}$$

(3) $$\begin{array}{c} VScore\left(M_{t},t\right)=\sum_{t=1}^{T}\frac{\sum_{u_{k}\in K\left(u_{t}\right)}{\left(r\left(u_{k},i\right)-\bar{r}\left(i\right)\right)}^{2}}{K}, \end{array}$$

where SQ is snapshot quality and data clustering quality is at time *t* and the methods are represented by rates variance. It is minimized the difference between the cluster items and maximized the similarity between the two clusters by difference the item scores into specific cluster at a specific timestep. In other words, internal similarity should be minimized and external distance should be maximized.

*VScore* is a variance score, which is represented the difference of any data from the mean of the *k* neighbors that rated to the item *i* at time interval *t*, in the matrix *M*_*t*_. *r*(*u*_*k*_, *i*) is the score of the *k* users in *n-th* neighbors to the item *i* at timestep *t*, which is in the matrix *M*_*t*_. $\bar{r}\left(i\right)$ is the mean of users rating in n-th neighbors to the item *i* at timestep *t*.

*M*_*t*_ is a matrix *n∗n*, which is showed the items entered at this timestep *t*. This matrix represented the relationship between each item pair and can be obtained on the basis of similarity or distance between two items at timestep *t*.

HC is defined using the normalized entropy criterion. Which stands for normalized mutual information (NMI), is defined by formula (4) in [1]:

(4) $$\begin{array}{c} NMI\left(t,t-1\right)=-2\frac{\sum_{i=1}^{C_{t}}\sum_{j=1}^{C_{t-1}}C_{ij}\log\left(C_{ij}N/C_{i}C_{j}\right)}{\sum_{i=1}^{C_{t}}C_{i}\log\left(C_{i}/N\right)+\sum_{j=1}^{C_{t-1}}C_{j}\log\left(C_{j}/N\right)}, \end{array}$$

where *C*_*t*_ is the numbers of clusters at timestep *t*, *C*_*t*−1_ is the number of groups at timestep *t* − 1, *C*_*i*_ is the sum of the elements *C* in the *i-th* row and *C*_*j*_ is the sum of the elements *C* in the *j-th* column and *N* denotes the number of nodes.

If *t* = *t* − 1, then *NMI*(*t*, *t* − 1)=1. The second goal is to increase *NMI*(*C*_*t*_, *C*_*t*−1_) at e.

HC deals with whether or not:

- Items that were in the previous clustering and are not in the new clustering
- Items that were not in the previous clustering and are in the new clustering

### 3.2. Evolutionary Clustering Based on Recommender Model

Now, the recommender modeling problem is formulated for predicting unknown ratings using the representation matrix and converted to an approximate weighted matrix. Evolutionary clustering has been used to solve this problem. Let *U*={*u*}_*u*=*i*^*n*^_ be a set of *n* users, and *I*={*i*}_*i*=1^*m*^_ is a set of *m* items. *A*=*n* × *m* is the scoring matrix, so that *a*_*ij*_ is the score of the user *u*_*i*_ for the item *i*_*j*_. There are two phases for the recommender model based on evolutionary clustering: neighbor calculation, which includes rating matrix clustering and calculates a specific user-item neighbor, which can be used later for prediction. Predicting consists the unknown rate estimation of neighbor.

Chaos motions are also characterized by the sensitivity of the early states; that small differences in the initial conditions can be rapidly amplified to produce large differences in response. The SA algorithm has a relatively slow convergence rate. The difference between chaotic applying and SA is the initialization and sequence of the chaos, rather than the Gaussian distribution, so that, in the small number of instances, a chaos pattern covered the search space better than the random distribution function. At the beginning of SA, all the samples are selected with the same probability and by reducing the temperature, the search space is limited and the chaos pattern characteristic is used [55, 59, 60].

### 3.3. Neighborhood Computation

The main purpose of this section is to calculate all the parameters needed to quickly predict an unknown rate. It is an evolutionary clustering method that basically involves calculating users and items clusters. First, it is selected the number of *K* clusters of the user, then consider the accuracy of the recommendation and resources required, also, it is performed evolutionary clustering on a user priority data. A model is created with an alternate user *k*, is derived directly from the *k* center {*c*_1_, *c*_2_, ..., *c*_*k*_} where each *c*_*i*_ is a vector of size *m*, means the number of items.

### 3.4. Prediction

In order to predict the *P*_*u*_*t*_,*i*_*t*__ rate, for the target user and item (*u*_*t*_, *i*_*t*_), the steps are performed. In the first step, the same user correlation with the target user is obtained with each of the alternative model user that are rated for personal use. The correlation coefficient is shown in formula (5) to find similar users to replace the target user [1]:

(5) $$\begin{array}{c} S_{u_{t},u_{k}}=\frac{\sum_{i\in I}\left(r_{u_{t,i}}-{\bar{r}}_{u_{t}}\right)\left(r_{u_{k},i}-{\bar{r}}_{u_{k}}\right)}{\sqrt{\sum_{i\in I}{\left(r_{u_{t,i}}-{\bar{r}}_{u_{t}}\right)}^{2}\sum_{i\in I}{\left(r_{u_{k},i}-{\bar{r}}_{u_{k}}\right)}^{2}}}, \end{array}$$

*i* is the set of items relevant to the target user and the *i-th* neighbor, and *r*_*u*_*t*_,*i*_ is the target user rate *u* for item *i* at timestep *t*. Such that ${\bar{r}}_{u_{t}}$ denoted target user *u* at timestep *t*. *r*_*u*_*k*_,*i*_ described *n-th* neighbor rates average to item *i* at timestep *t*. ${\bar{r}}_{u_{k}}$ averaged *n-th* neighbor rates at timestep *t*.

In the second step, prediction is adjusted by using weighted average that formulated in (6):

(6) $$\begin{array}{c} P_{u_{t},i_{t}}={\bar{r}}_{u_{t}}+\frac{\sum_{n=1}^{K}\left(r_{u_{k},i}-{\bar{r}}_{u_{k}}\right)*S_{u_{t},u_{k}}}{\sum_{n=1}^{K}S_{u_{t},u_{k}}}, \end{array}$$

*r*_*u*_*k*_,*i*_ denoted the *n-th* neighbor rate to item *i* at timestep *t*, ${\bar{r}}_{u_{k}}$ described the average rate of *n-th* neighbor at timestep *t*, ${\bar{r}}_{u_{t}}$ is the mean rate of target user at time *t*, *S*_*u*_*t*_,*ci*_ is the similarity of the target user and *n-th* neighbor at timestep *t* and *k* is the number of neighbors at timestep *t*.

After creating the prediction matrix, it is sorted in descending order and based on the Top-N algorithm, recommends *N* best items to the user, and then the evaluation criteria is calculated. In memory-based recommender systems, users can usually evaluate their resources, and recommenders can suggest items based on the most recent items they have already rated to improve the quality of the suggestions.

The proposed method is performed in four phases:

1. Evolution of clusters by Memetic algorithm
2. Calculate Neighbors
3. Prediction
4. Recommendation

## 4. Proposed Method Evaluation

As a recommender system, for a target user *i*-th, is predicted all nonrated items and is proposed the highest-rated items. In order to evaluate recommender algorithms, the data are usually divided into two parts: training set *E*^*T*^ and search set *E*^*p*^. The training set is behaved as known information, while none of the search set information is allowed to recommend. In this section, evaluation is used four criteria of accuracy, recall, mean absolute error and F-measure.

### 4.1. Criteria Used to Evaluate the Proposed Method

In order to measure the predictions, the absolute mean error (MAE) is used, which is widely used in various algorithms to measure statistical accuracy. The absolute mean error for the user *U* is calculated by formula (7):

(7) $$\begin{array}{c} MAUE\left(U\right)=\frac{\sum_{i\in I}\left|A_{u,i}-p_{u,i}\right|}{\left|I_{u}\right|}, \end{array}$$

*I*_*u*_: Number of items the user *u* has been rated, (*A*_*u*,*i*_, *P*_*u*,*i*_): User *u* actual and predicted rates are in the test set.

Finally, the absolute mean error for all users in the dataset is calculated by the (8):

(8) $$\begin{array}{c} MAE=\frac{\sum_{u=1}^{K}MAEU\left(U\right)}{K}. \end{array}$$

Precision and Recall criteria are commonly used in evaluation in information retrieval systems. Details of these two parameters are shown in Table 1.

The item set must be categorized into two classes: “relevant” and “irrelevant.” In the dataset used in the proposed system where scores are 1 to 5. The rates 1 to 3 are considered as irrelevant items and rates 4 and 5 are considered as related items. For example, *N*_*r*,*s*_ shows a set of items that are available in two sets of recommendations as a related user and item.

The Precision is the ratio of proposed items to the total proposed items and is defined:

(9) $$\begin{array}{c} \mathrm{precision}=\frac{N_{r,s}}{N_{s}}. \end{array}$$

The Recall is the ratio of related proposed items to all relevant items and is defined:

(10) $$\begin{array}{c} \mathrm{recall}=\frac{N_{r,s}}{N_{r}}. \end{array}$$

As the number of suggestions increases, precision decreases and Recall increases, these two parameters are combined in formula (11) and as F1 criterion is defined.

(11) $$\begin{array}{c} F_{1}=\frac{\mathrm{2\times precision\times recall}}{\mathrm{precision+recall}}. \end{array}$$

### 4.2. Experimental Results

#### 4.2.1. Dataset

This dataset contains 100,000 rates, given by 943 users to 1682 films (the item-user matrix consists of 943 rows and 1682 columns). This bipartite network consists of 100,000 user movie ratings, which can be accessed from https://grouplens.org/datasets/movielens/100k/. This dataset is a stable benchmark dataset which is used in this experimental result.

#### 4.2.2. Proposed Method Evaluation

**Figure 3:**
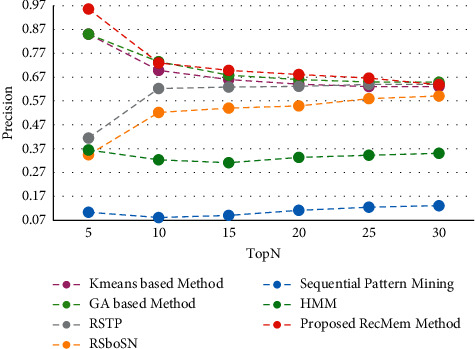
Precision value of the RecMem method compared to other methods.

**Figure 4:**
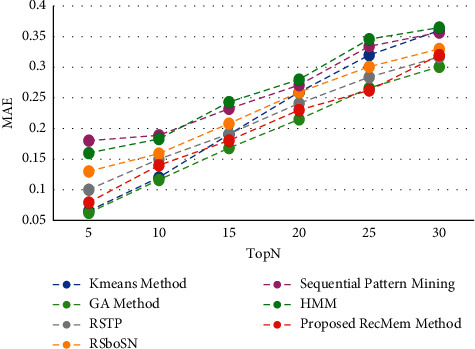
MAE value of the RecMem method compared to other methods.

**Figure 5:**
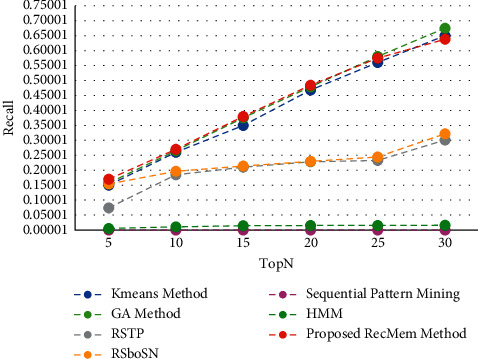
Recall value RecMem method compared with other methods.

**Figure 6:**
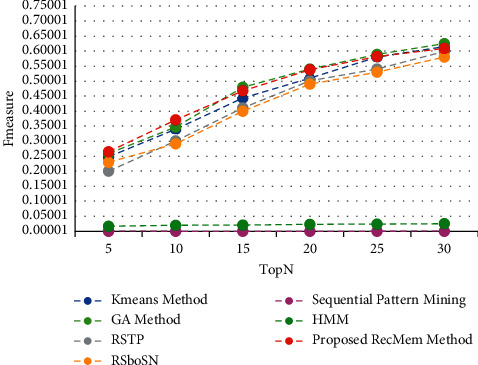
F-measure value RecMem method compared with other methods.

The time is a crucial parameter to users who are change their interest over time. From our point of view, users are two categories whom their interest may change over time by being older, the other group are whom must use the specific subject for a short time that it has several causes such as personal project, friend request. In this paper, the users interest considered to predict user needs over time. One of the disadvantages of this is that it does not focus on more final recommendations. This work is very accurate in the first five suggestions, but with increasing the number of recommendations, the precision of the work decreases. This means that system performance diminishes as the number of suggestions grow. At first, it uses a combination of two algorithms to evolve user clusters over time. By doing this, each user will be in the right cluster according to her interest that changes over time. Then is developed by using the chaotic parameter and improves the results. To demonstrate novelty, experiments have been performed on the Movielens 100 k dataset to show clustering based on it. All results are run on 150 iterations in evolutionary algorithm. It is evaluated the accuracy, absolute error mean (MAE), recall and F-measure value of the proposed method in the conventional K-means clustering based method [2], Time-Aware Clustering enriched with Genetic algorithm [1], proposed RecMem method, Sequential Pattern Mining method [27], Markov Hidden Model method [27], RSTP method [25] and RSboSN method [25] which are shown in Figures 3–6 respectively. As it can be seen in Figure 3, the proposed RecMem method have the highest accuracy compared to other methods. The precision of the system in the first five recommendations is 0.95 and by increasing number of recommendations, the precision reduces slightly. However, according to the results, the accuracy of the proposed method is still better than the other methods. As it mentioned before, one of the drawbacks is that as the number of suggestions increased, the accuracy slightly decreased. Also, in Figure 3, the Kmeans, GA and proposed RecMem methods have a similar behavior. Initially, the Kmeans and GA methods have precision about 0.88 and the RecMem method is about 0.95. But as the number of items increases, the precision of the RecMem method decreases slightly to about 0.70. The RSTP, RsboSN methods have precision about 0.40 in the beginning and by increasing the recommendations number is about 0.59. The Sequential Pattern Mining and HMM methods have no changes through the incremental suggested items. The Sequential Pattern Mining is about 0.37 and HMM is about 0.07 during the iterations. As shown in Figure 3, the proposed method performance is very good, specially in 5 tops. The MAE is a measure of error between paired observations expressing the same phenomenon. Given that accuracy is decreased by increasing number of suggestions, it is normal for MAE to increase. As it shown in Figure 4, the proposed methods have a low mean absolute error (MAE) close to the two ordinary K-means clustering based method [2] and Genetic Algorithm based method [1].

The recall parameter is the fraction of total amount of relevant items that were actually retrieved. In the initial results of the Top-N algorithm, which starts with five suggestions, the recall level is low, which has increased in the proposed RecMem, GA and K-means methods with increasing the number of recommendations, but in HMM and Sequential pattern mining methods has not changed much. Also, RSTP and RSbonSN methods have had very little increase. As it shown in Figure 5, the Recall values for all methods are growing like the RecMem. The evaluation parameter of the F-measure is a combination of accuracy and recall, as shown in Figure 6.

The numeric results shown in Table 2 which considered the 5-Top. As results in Table 2, the RecMem method perform much better than Sequential Pattern Mining method which performs clustering sequentially [27]; it also performs more accurate than the hidden Markov model [27], RSboSN method [25] and RSTP method [25]. Although conventional K-means clustering-based method [2] and Genetic Algorithm clustering-based method [1] have an accuracy about 0.85, the proposed method has higher accuracy.

#### 4.2.3. Evaluating the Impact of RecMem Performance on Cold-Start Items Problem

**Figure 7:**
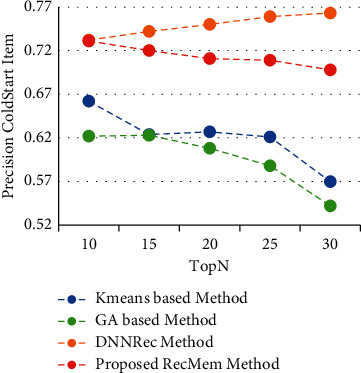
Precision value of RecMem method in the cold-start item compared with other approaches.

**Figure 8:**
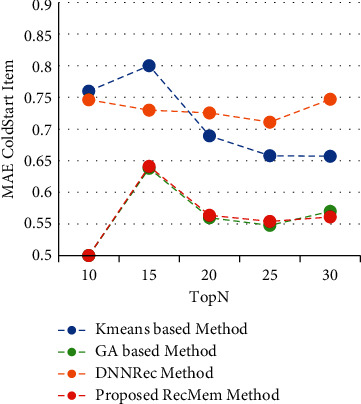
MAE value of RecMem method in the cold-start item compared with other approaches.

**Figure 9:**
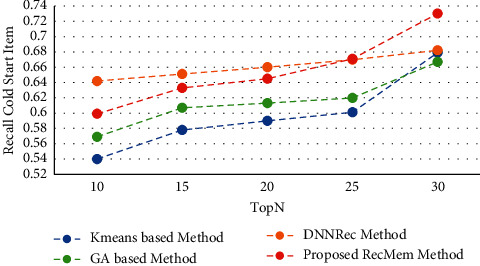
Recall value RecMem method in cold-start item compared with other approaches.

**Figure 10:**
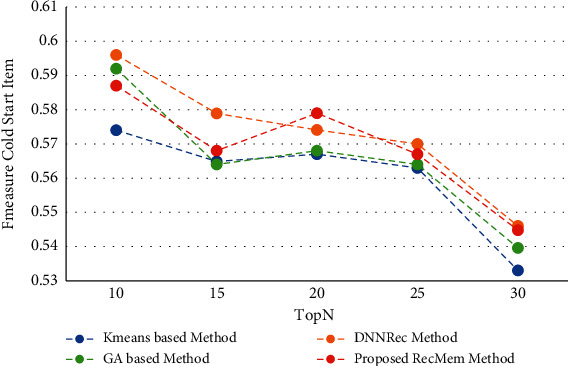
F-measure value RecMem method in cold-start item compared with other approaches.

In this section, the RecMem is evaluated the cold-start item approach. As it mentioned in previous parts, cold-start items describe how new items are added to the item category. These items have not had any rates in the beginning and the items attributes is the only information available. The results of experiments performed to assess the RecMem method in dealing with the issue of cold-start items are stated. It should be noted, however, that the MovieLens dataset is also more common than other datasets used to evaluate recommender systems, and in the MovieLens dataset, noncold start items should have at least twenty rates. Accuracy, Absolute Mean Error, Recall and F-Measure of conventional K-means Clustering Method [2], Time-Aware Clustering-based method enriched with Genetic Algorithm [1], the RecMem with the item cold-start approach and DNNRec method [30] are shown in Figures 7–10, respectively.

As it shown in Figure 7, the RecMem and DNNRec methods for the cold-start problem have a prediction accuracy of about 0.7 at first. Increasing the number of recommended items is reduced the accuracy of the prediction, with the exception of DNNRec method. The DNNRec precision is increased by rising the offered items which is about 0.76. The accuracy of RecMem method is decreased a little to 0.70 by rising the number of suggested items. The Kmeans and GA methods is started at about 0.65 which are decreased by increasing suggested items. As it mentiond, although, the RecMem method has a fair result in cold-start item, but it can be improved by rising the recommendations items. Due to Figure 8, the RecMem method has very low mean error, which means that an accuracy about 0.5 in the cold start item makes prediction by GA method with a very low mean error. According to the MAE results are shown in Figure 8, the RecMem approach and the GA method have the least mean error in the cold-start item compared to other methods. The RecMem method have a good recall performance by contrast to other methods with increasing number of recommendations. As shown in Figure 9, the recall increasing through the recommended item raised. Comparing the cold-start problem of the RecMem method in cold start item with the DNNRec method [30], it is shown that the accuracy of the proposed method is close to the DNNRec method [30], but the MAE of the RecMem method is shown in Figure 8, that is lower than other methods. The F-measure evaluation parameter achieved from the combination of accuracy and recall that it shown in Figure 10.

#### 4.2.4. Evaluating the Impact of RecMem Performance on Cold-Start User Problem

**Figure 11:**
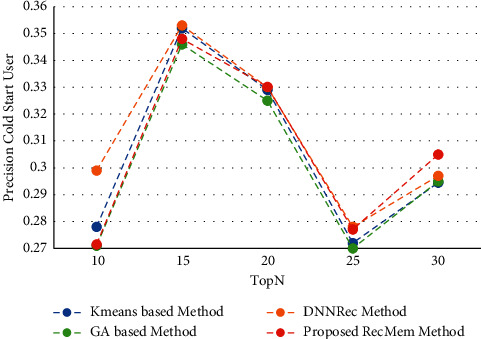
Precision value of RecMem method in the cold-start user compared with other methods.

**Figure 12:**
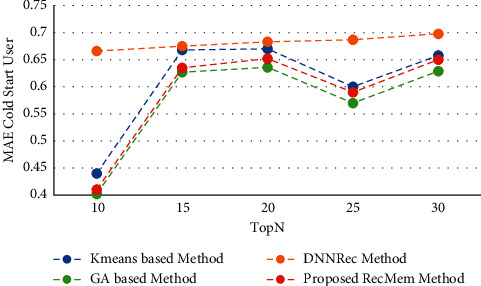
MAE value of RecMem method in the cold-start user compared with other methods.

**Figure 13:**
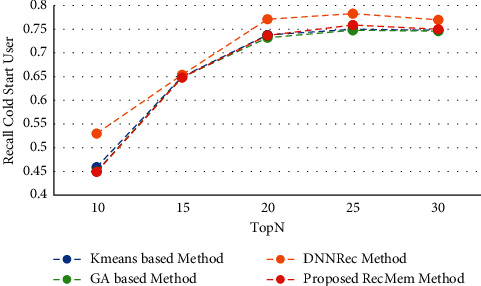
Recall value of RecMem method in the cold-start user compared with other methods.

**Figure 14:**
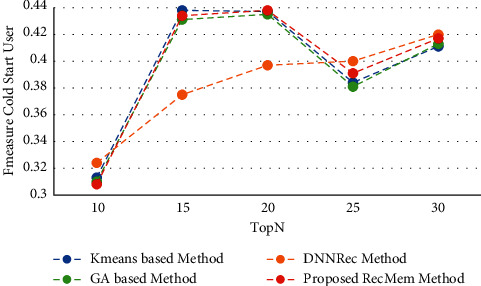
F-measure value of RecMem method in the cold-start user compared with other methods.

This section shows the experiment results to evaluate the RecMem approach to address the problem of new users. Accuracy, MAE, Recall and F-Measure parameters based on conventional K-means clustering methods [2], Time-aware clustering enriched with Genetic Algorithm [1], proposed RecMem method with cold-start user approach and DNNRec method [30] is illustrated in Figures 11–14, respectively. As shown in Figure 11, the RecMem method is performed as same as the exist methods which achieved the peak of accuracy in 15 suggestions at 0.35. In contrast to 30 offered users, the RecMem obtains precision about 0.31 which carried out superior than others. Due to Figure 12, the MAE of the RecMem method is close to the K-means [2] and GA [1] methods. The recall parameter results are shown in Figure 13, which increased by raising the suggested users. The F-measure evaluation parameter achieved from the combination of accuracy and recall that it shown in Figure 14.

The cold-start user experiments are proved that the RecMem approach and Genetic Algorithm have low MAE. In the case of the cold-start user problem, the RecMem in the number of recommendations lower than 30 have the same precision to other methods, but by increasing the number of suggestions, the accuracy is better than other methods. In the RecMem, as the number of recommendations is increased, the recall for all methods is close to each other. As it is demonstrated in results, the RecMem method has not been performed as well in cold-start problem. There is an opportunity for the future researchers to improve the system to extend the quality of system performance for cold-start problems.

## 5. Summary and Conclusion

In today's e-commerce world, recommender systems are the most powerful tool for personalizing customer information. These systems help e-shops only display information to the user that they are interested in. The most common method is collaborative filtering that can be divided into user-based method and item-based approach. In the user-based collaborative filtering approach, to decide whether to offer an item to an active user, users who have already shared the item with the active user also evaluate the item. It is then using the rate that users have given to the target item to predict the rate, which the user is likely to give. Finally, if the predicted rate indicates the active user's interest in the target item, the target item is proposed. By using such a method in a recommender system, there will be users who use the system more than other users in terms of their ratings and thus have a greater impact on those systems. These users are referred to as influencers in recommender systems. In the collaborative filtering approach, the user-item matrix is first analyzed to identify the relationships between different items and then these relationships are used to calculate suggestions to users. The contribution of the proposed method to the recommender systems means that an evolutionary algorithm is used to investigate the evolution process of a system. This method can be used in collaborative filtering of recommender systems to integrate new data over a period of time and update user specifications to its current state. This paper investigates the performance of the evolutionary clustering algorithm and generates high quality clusters and predicts suggestion based on the actual Movielens dataset. The evolving cluster is shown user priority and is created a user's favorite recommendation. The RecMem method is used a scale called variance to compute the user ratings difference and thus calculate the evolutionary trend change at each timestep. Therefore, the algorithm results in the cluster being updated at each time interval.

Experimental results in the large real-world dataset show that this algorithm can provide high-quality predictions over traditional clustering algorithms and other model-based approaches. The biggest disadvantage of model-based methods is that their training process is slow and has been significantly improved in the proposed method of this study. The proposed method can be easily applied in another domain as well. The RecMem method is a recommender system that provides accurate results and a gradual and without time-consuming update. One of the major challenges in implementing a user-based algorithm is the subject of cold-start item and user in the rates matrix. The origin of this problem is primarily users who are new to the system and the system has no information about them and cannot offer accurate information, as well as items that are new to the system and have not yet been rated. In this study, the proposed method for the cold-start problem which the challenge is resolved with a relatively low. While in the RecMem method, using clustering of users and items also leads to improved prediction and consequently suggenstion increased accuracy. The demerits of proposed method in this paper, the lower precision is achieved when the number of recommendations increasing. Also, the precision of cold-start problem is achieved unsatisfied level. The computational time depends on number of iterations which the evolutionary algorithm is running.

## 6. Future Works

In recommender systems, a wide range of subjects must be considered. In addition to the solution adopted and described in this paper, there are many ideas that we have not focused on. The approaches proposed in this paper do not provide the high quality of cold-start problems that can be done by researchers in future works. On the other hand, there are some limitations in this work, which researchers can overcome in future work and are as follows:

- Using matrix analysis methods to implement dynamic feedback entities over time
- Consider the proposed method on other datasets for generalizing
- Using other evolutionary algorithms to optimize the proposed algorithm
- Using ontology for semantic contents to improve the prediction accuracy
- Extending proposed method by using multiobjective evolutionary algorithms

The list mentioned above can be used by future researchers to improve and develop the approach presented in this paper.

## Figures and Tables

**Table 1:** The items into two relevant and irrelevant categories for calculating precision and recall.

	Selected	Not selected	Total
Relevant	*N*_*r*,*s*_	*N*_*r*,*n*_	*N*_*r*_
Irrelevant	*N*_*i*,*s*_	*N*_*i*,*n*_	*N*_*i*_
Total	*N*_*s*_	*N*_*n*_	*N*

**Table 2:** Two proposed methods Precision and Recall compared with other methods.

Method based on	Fmeasure- 5Top	Precision- 5Top	Recall- 5Top
Proposed RecMem	0.265	0.956	0.17
GA	0.259	0.85	0.17
K-means	0.248	0.85	0.15
RSboSN	0.23	0.344	0.154
RSTP	0.2	0.4148	0.074
HMM	0.01664	0.3644	0.0054
Sequential pattern mining	0.0000712	0.103	0.0000356

## Data Availability

The MovieLens 100K data set used to support the findings of this study have been deposited in the GroupLens repository https://doi.org/10.1145/2827872.
